# Attitudes towards Prosthodontic Clinical Decision-Making for Edentulous Patients among South West Deanery Dental Foundation Year One Dentists

**DOI:** 10.3390/dj4020012

**Published:** 2016-05-10

**Authors:** Andrew Barber, James Puryer, Sam Leary, Lisa McNally, Dominic O’Sullivan

**Affiliations:** 1 Cambridge University Hospital, Cambridge Biomedical Campus, Hills Road, Cambridge CB2 0QQ, UK; andrew.barber@addenbrookes.nhs.uk; 2 School of Oral and Dental Sciences, Bristol Dental Hospital, Lower Maudlin Street, Bristol BS1 2LY, UK; l.m.mcnally@bristol.ac.uk; d.j.osullivan@bristol.ac.uk; 3 Bristol Nutrition (Biomedical Research Unit), School of Oral and Dental Sciences, Bristol Dental Hospital, Lower Maudlin Street, Bristol BS1 2LY, UK; s.d.leary@bristol.ac.uk

**Keywords:** dentist, decision making, prosthetics, dentures

## Abstract

The aim of this study was to describe Dental Foundation year one dentists’ attitudes towards prosthodontic decision making for edentulous patients, and identify whether there are gender differences in these attitudes. All South West Deanery trainees were invited to take part in the study between May and June 2011 and a previously piloted questionnaire was administered to the trainees by their training programme directors. The questionnaire posed questions based upon a clinical scenario of discussing treatment options with patients. Seventy-two questionnaires were used in the analysis (91% overall response rate). Trainees perceived their own values to be less important than the patient’s values (*p* < 0.001) in decision making, but similar to the patient’s friend’s/relative’s values (*p* = 0.1). In addition, the trainees perceived the patient’s values to be less important than their friend’s/relatives (*p* < 0.001). Sixty-six per cent of trainees acknowledged an influence from their own personal values on their presentation of material to patients who are in the process of choosing among different treatment options, and 87% thought their edentulous patients were satisfied with the decision making process when choosing among different treatment options. Fifty-eight per cent of trainees supported a strategy of negotiation between patients and clinicians (shared decision making). There was no strong evidence to suggest gender had an influence on the attitudes towards decision making. The finding of a consensus towards shared decision making in the attitudes of trainees, and no gender differences is encouraging and is supportive of UK dental schools’ ability to foster ethical and professional values among dentists.

## 1. Introduction

Decision making is an essential part of all healthcare delivery. Clinicians need to appraise a wide range of factors prior to arriving at a decision that represents optimal care for their patient. These factors include clinical factors, patient values, the available research evidence, clinical guidelines, their previous clinical experience and medico-legal implications. The difficulty for patients, their accompanying persons and clinicians of integrating the available information in order to make the optimal treatment choice is increasingly recognised.

Three models of the clinician-patient relationship have been described [1]:

⚫ Paternalism (traditionally the clinician makes decisions for the patient);
⚫ Consumerism (primarily based upon patient preferences); and
⚫ Shared decision making (whereby a consensus is reached).

UK legislation dictates that pursuing a strongly paternalistic decision making style could leave a dentist vulnerable to the medico-legal challenge of failure to obtain valid consent for treatment [2]. Not fully considering patients’ wishes as part of the decision making process and making judgements purely on technical factors, even if the decision is evidence based, represents a failure to respect the ethical principles of choice and free will, which are central to patients’ autonomy. However, following a consumerist decision making style could lead to situations where the patient requests treatment that is at odds with what the clinician believes is in the patient’s best interest [3].

Shared decision making (SDM) allows both parties to play an active role in the decision making process and arrive at a decision through a negotiation [4]. A systematic review [5] of the effectiveness of SDM concluded that despite the considerable interest in applying SDM clinically, there is little research regarding its effectiveness. SDM is particularly suitable for long-term decisions [5], especially in the context of a chronic illness, and when the intervention contains more than one session. The edentulous state (loss of all teeth) is a chronic condition [6] and prosthodontic interventions will inevitably require multiple treatment sessions and long term care. The SDM concept seems appropriate in such cases. Although there is little evidence that a dentist’s gender has a role in their choice of decision making style or the patient–dentist relationship [7], it has been reported that female doctors show a greater affinity for collaborative models of patient–physician relationship than do their male colleagues [8].

Treatment options for the edentulous patient include no treatment, conventional complete dentures, implant retained overdentures and implant supported fixed bridgework. Involving edentulous patients in prosthodontic decision making is essential due to the diverse range of functional outcomes [9], risk of complications and costs [10] associated with the various therapies.

The amount of clinical decision making experience that undergraduates develop in undergraduate degree courses varies considerably depending on curriculum design [11]. In more traditional dental school environments, prosthodontic options may have been decided before the student sees the patient for a particular type of treatment or prosthesis. In other styles of undergraduate dental education based in primary care settings [12], students may become involved in prosthodontic management decision making more readily, although in a supervised fashion.

The majority of newly qualified dentists in the United Kingdom (UK) enter Dental Foundation Training (DFT) directly after graduation. There are seventy-four DFT schemes in total, located in twelve deaneries across England and Wales. There are a total of seven dental foundation training programmes in the South West dental postgraduate deanery: Bath, Bristol, Exeter Plymouth, Salisbury, Taunton and Truro. Each of these programmes has a training programme director who arranges the study days for the trainees and acts as an initial point of contact between the trainees and the dental postgraduate deanery.

The purpose of DFT is to enhance clinical and administrative competence and promote high standards through relevant postgraduate training including National Health Service (NHS) general dental practice. The DFT curriculum states the ability to “Demonstrate effective and ethical decision making” as one of the major competencies required as part of the professionalism domain [13]. To our knowledge, decision making practices amongst trainees has not yet been explored in the dental literature.

Therefore, the aim of this study was to identify the South West deanery trainees’ attitudes towards prosthodontic decision making for edentulous patients, and assess for gender differences.

## 2. Methods

### 2.1. Questionnaire Development

**Figure 1:**
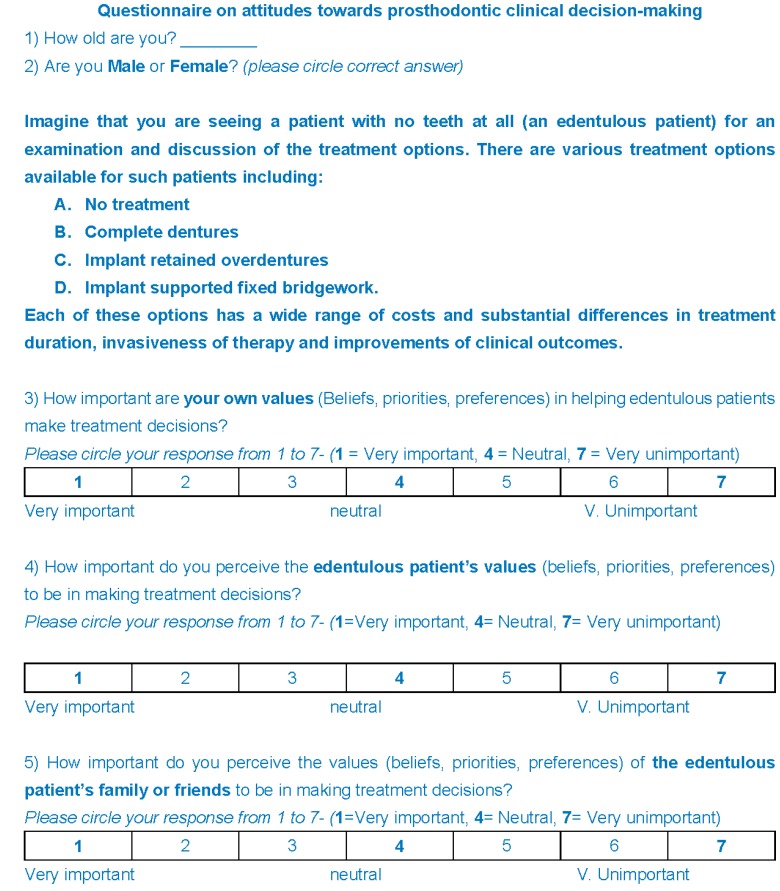

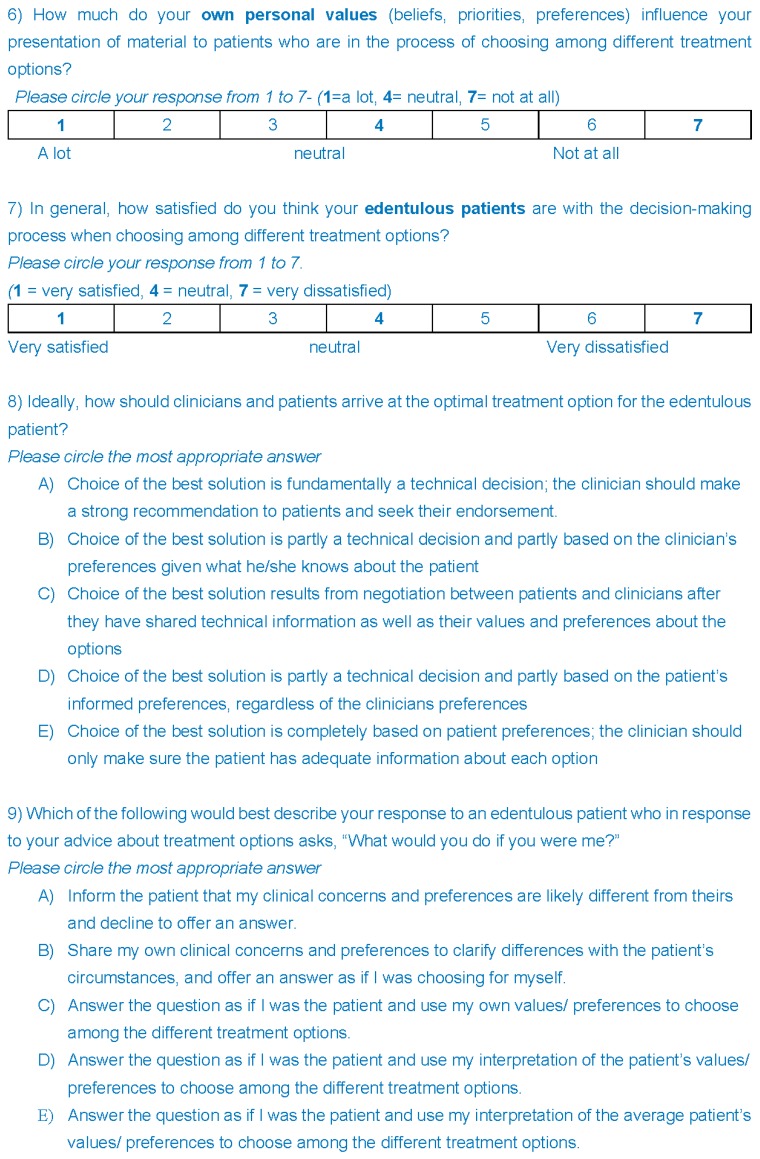
The questionnaire used in the survey.

A questionnaire (Figure 1) was developed to assess the attitudes and beliefs of trainees towards prosthodontic decision making for the edentulous patient, based on a literature review of published peer reviewed studies into clinical decision making in prosthodontics [13]. The first two items asked about age and gender. Then a short clinical scenario of the discussion of treatment options with an edentulous patient was posed. Five questions then followed relating to the influence of practitioner and patient values towards treatment planning for an edentulous patient; possible responses were between 1 (very satisfied) and 7 (very dissatisfied). The final two questions related to the participant’s beliefs about the optimal way of approaching clinical decision making; responses to both questions were one out of five possible options.

Pilot questionnaires were administered to four final year dental students and two general practitioners, and the questionnaire content was then revised. Full ethical approval for the study was obtained from the University of Bristol, Faculty of Medicine and Dentistry Committee for Ethics.

### 2.2. Study Sample

All trainees (*n* = 79) enrolled on the South West deanery scheme were selected as the study population, and the South West Postgraduate Dental Dean granted approval to approach the trainees. Study recruitment was co-ordinated with the co-operation of the South West region’s seven training programme directors. Each programme director was contacted via email and letter and given full details of the study including copies of the participant information sheet and the study questionnaire. Follow up telephone calls and face-to-face meetings were made by one investigator to answer any queries about the study and ensure the method of administering the study was clearly understood. A suitable date to administer the study questionnaire was selected for each training scheme within May and June 2011. The trainees were then sent an e-mail inviting them to participate in the study seven days before it was administered, which gave sufficient time for a valid informed consent process for participation. A stamped addressed envelope was also included for return of the questionnaires. On the date of administration of the study, a brief verbal summary of the study was given by the training programme directors to the trainees, based on the participant information sheet. The participant information sheet and the study questionnaire were given to the trainees and they were given sufficient time to consider fully their choice to participate in the study and to complete the questionnaire if willing.

Following completion of the questionnaire, the participants were asked to place their form anonymously in a collection box. The training programme director then collected the forms from the box and returned them to the principal investigator in the stamped addressed envelope along with any incomplete questionnaires from those who declined to participate in the study.

### 2.3. Statistical Analysis

The responses to each of the nine questions were summarised by proportions or median with range/inter-quartile range (IQR) as appropriate. Wilcoxon signed rank tests were used to compare the trainees’ perceptions of the relative importance of the dentist’s, patient’s and patient’s relative/friend views. Mann Whitney U tests were used to assess gender differences. All analysis was undertaken using SPSS 16.0 for Windows (Release 16.0.2).

## 3. Results

Of the 79 questionnaires that were distributed, five were not returned and two were returned blank; this allowed 72 (91% of those distributed) to be used in the analysis. The median age of respondent was 24 (range = 23–42) years, and 38% were male and 62% female.

**Figure 2:**
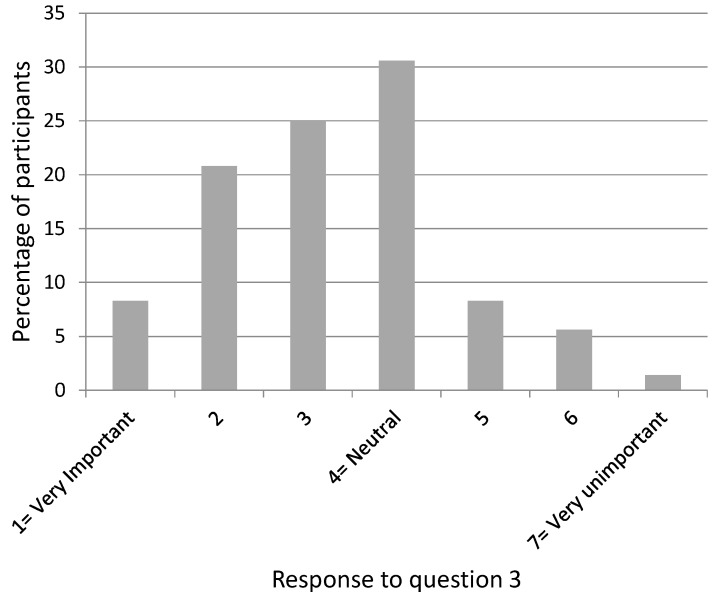
Participants’ response to Question 3: “How important are your own values (beliefs, priorities & preferences) in helping edentulous patients make treatment decisions?”

**Figure 3:**
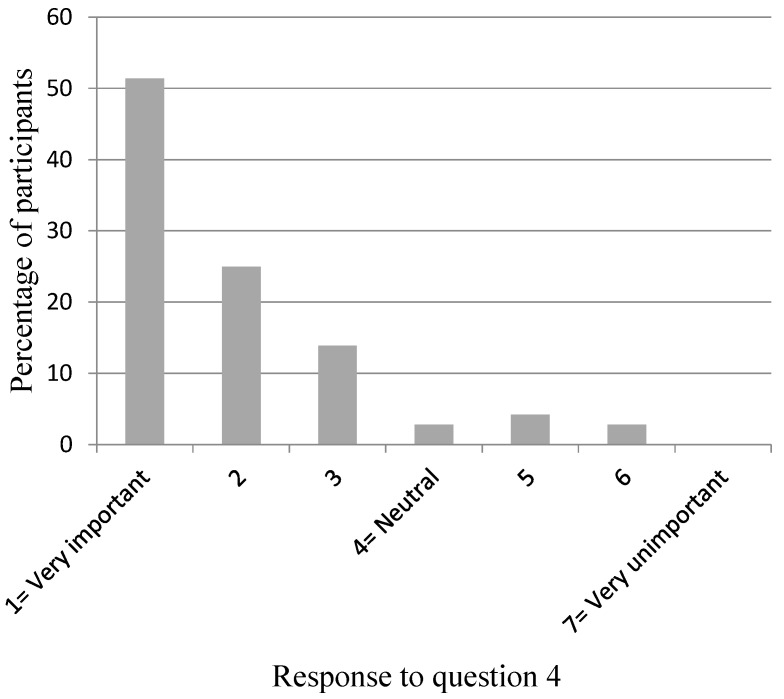
Participants’ response to Question 4: “How important do you perceive the edentulous patient’s values (beliefs, priorities, preferences) to be in making treatment decisions?”

**Figure 4:**
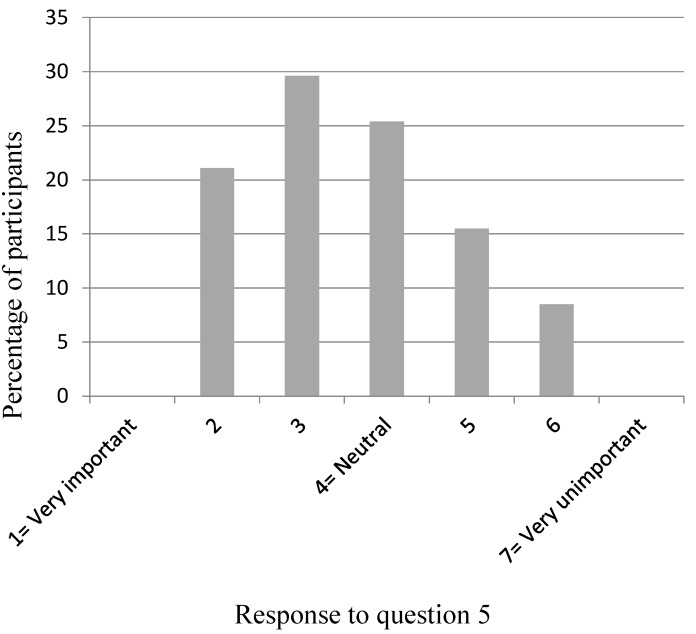
Participants’ response to Question 5: “How important do you perceive the values (beliefs, priorities, preferences) of the edentulous patient’s family or friends to be in making treatment decisions?”

**Figure 5:**
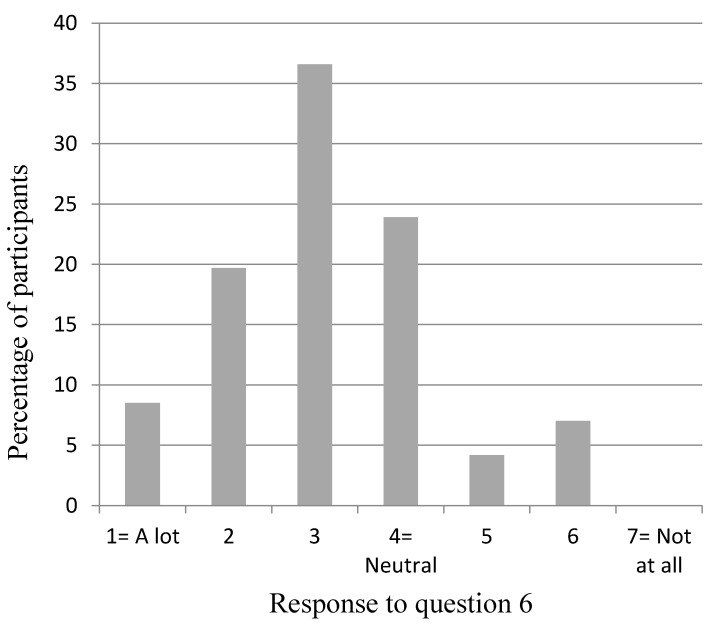
Participants’ response to Question 6: “How much do your own personal values (beliefs, priorities & preferences) influence your presentation of material to patients who are in the process of choosing among different treatment options?”

**Figure 6:**
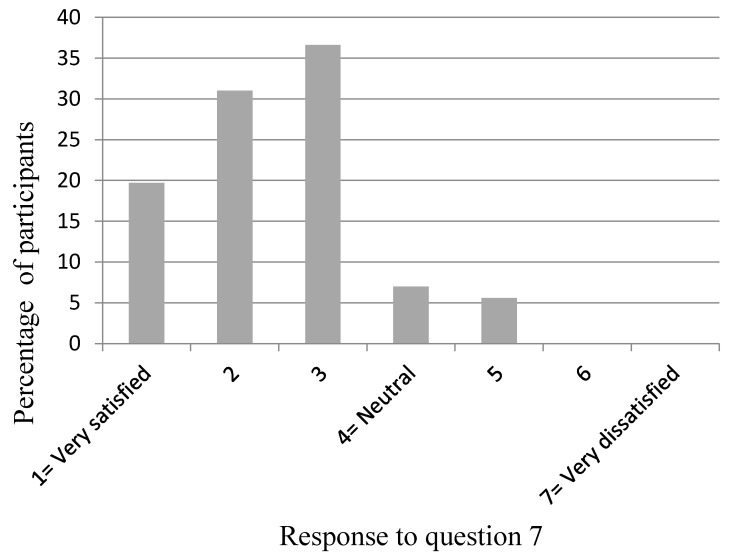
Participants’ response to Question 7: “In general, how satisfied do you think your edentulous patients are with the decision-making process when choosing among different treatment options?”

Figure 2, Figure 3, Figure 4, Figure 5 and Figure 6 show the whole group summary of category percentages for Questions 3 to 7, while Table 1 (column 1) shows the median (IQR) values for these questions. With respect to the relative importance of the dentists’, patient’s, and patient’s friend’s/relative’s values in decision making, the trainees perceived their own values to be less important than the patient’s values (*p* < 0.001), but similar to the patient’s friend’s/relative’s values (*p* = 0.1). In addition, the trainees perceived the patient’s values to be less important than their friend’s/relatives (*p* < 0.001). Sixty-six per cent of trainees acknowledged an influence from their own personal values on their presentation of material to patients who are in the process of choosing among different treatment options. Eighty-seven per cent of trainees thought their edentulous patients were satisfied with the decision making process when choosing among different treatment options. Table 2 (column 1) shows the trainees’ opinions on how treatment options should be decided upon, with the highest percentage (58%) supporting negotiation between patients and clinicians. Table 3 (column 1) shows the trainees’ responses to being asked “what would you do if you were me?”, with the highest percentage (38%) offering an answer as if the trainee was choosing for themselves.

**Figure 7:**
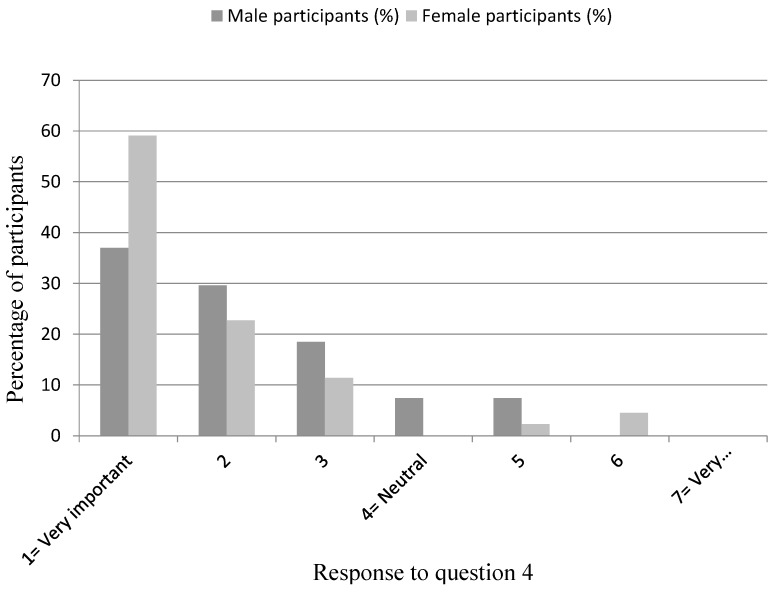
Male and female participants’ response to Question 4: “How important do you perceive the edentulous patient's values (beliefs, priorities, preferences) to be in making treatment decisions?”.

Table 1 shows the median (IQR) responses to Questions 3 to 7 in males (column 2) and females (column 3) separately. There was no statistical evidence for gender differences for Questions 2 or 5 to 7 (see column 4 for *p*-values). There was however, weak evidence that males perceive less importance of the edentulous patient’s values in decision making compared to their female counterparts (Figure 7). Table 2 and Table 3 show the responses in males (columns 2) and females (columns 3) to Questions 8 and 9, with 59% (M)/57% (F) and 41% (M)/36% (F) giving the most popular response to Questions 8 and 9, respectively.

## 4. Discussion

The aim of the current study was to describe South West deanery year one foundation dentists’ attitudes towards prosthodontic decision making for edentulous patients and identify any gender differences. The dental foundation training curriculum states the ability to “Demonstrate effective and ethical decision making” as one of the major competencies required as part of the professionalism domain [14], and we are not aware that decision making practices amongst trainees has been explored in the dental literature. The decision to survey all South West trainees (a census) rather than utilising a representative sample, reduced selection bias and increased the validity of the study, and using a questionnaire that has been thoroughly piloted and tested has been associated with increased response rates [15]. The 91% overall response rate obtained in the study, is higher than that achieved for the majority studies involving dentists [16]. In this study, the questionnaire was based on the literature review that identified a previously developed and published instrument [13]. Closed questions were used and it is acknowledged that with such designs the richness of responses can be lower [17]. Such a design was necessary however, in order to generate quantitative data that would address the aims of the study.

The Likert scale [18] is a summated rating scale and is commonly used to assess attitudes [19]. The Likert scale does not measure the attitude per se [20], but in this study allowed the comparison of survey items, for example, the participants’ perceptions of the relative importance of dentist’s, patient’s and relatives’ values in decision making.

Questionnaire research can never be completely objective [21]. The questionnaire was intended to give an insight into the psychological perspective and attitudes of the trainees towards the decision making process with edentulous patients, not assessing the actual clinical practises of dentists.

The respondents’ anonymity was protected, and this was made clear to potential participants. This helps reduce method bias and increases validity especially at the judgement and response editing or reporting stages [22]. The respondents were also reassured in the participant information sheet that the questionnaire was “not a test and there are no right or wrong answers”. This was designed to strengthen the study’s validity and reduce response bias by reducing participants’ evaluation apprehension (anxiety about being scrutinised) and make them less likely to edit their responses to show behaviour that would be expected of them [22].

The median age of 24 years is unsurprising given the most common age of entrance into the five year BDS programme is at 18 years and that the dental foundation year one takes place usually directly after dental school. A study examining all UK dental undergraduate admissions found that more than 90% entered university aged less than 21 years [23]. The significance of a dentist’s age in prosthodontic decision making has not been explored in the dental literature. Edentulism affects 6% of the UK population, but only 1% of adults between 45 and 54 years are edentate compared to 47% of those aged 85 years and over [24]. Therefore, the demographic of the typical edentulous patient is greatly different from that of the dental foundation trainees studied. This age gap could affect the ability of trainees to empathise with edentulous patients and might also mean that it is unlikely they would share similar values with respect to choosing the most appropriate treatment option.

The gender distribution of the trainees is typical of the gender distribution among UK dental undergraduates [23] with a slightly higher proportion of females [25]. The proportion of females was significantly greater (*p* < 0.001) than the 16% observed among the group of North American prosthodontists previously studied [16].

The results demonstrate that the dentists rated the edentulous patient’s values as more important than either their own values or those of the patient’s family or friends, and that their own values and those of the edentulous patient’s family or friends had only neutral or slight importance in helping edentulous patients make treatment decisions. This suggests that the principle of shared decision making or even a consumerist model is supported. The order of the two most popular choices by patients of their preferred role in decision making [26] matches exactly the two most common choices by trainees on what constitutes the best way of arriving at the optimal treatment option. This is encouraging since shared decision making, with patients taking a collaborative decisional role, has been shown to be the preferred model of decision making by patients in both a primary care and secondary care dental setting [26]. The concept is also in line with the medico-legal requirements of obtaining informed, valid consent to treatment and respecting patient’s autonomy. Dentists have the responsibility to ensure that patients have had the best opportunity to be involved in decision making about the care of their bodies [27] and the views of DF1s were in line with this notion.

Regarding how the trainees present material to patients, in addition to what is actually said, the eyes, face, posture and gestures form a package of non-verbal communication that can affect the perceptions of others [28]. These influential changes in voice and behaviour may be conscious or subconscious. The responses to Question 6 are potentially indicative of trainees supporting the concept of shared decision making and respecting patient autonomy.

As most dentists considered their patients to be satisfied with the decision making process, a high level of confidence in discussing treatment options is suggested. Undergraduate students’ confidence in dentist–patient interactions has been shown to be related to how well students felt they were taught and how often they encountered the situation [29]. One assumption that has been made is that participants have indeed had adequate training in these skills, and that they have treated a sufficient number of edentulous patients throughout their undergraduate career in order to form these opinions. A previous study [30] found new UK graduates entering vocational training with little confidence in denture techniques and unable, sometimes unwilling, to undertake these procedures. A later UK survey [31] suggested that dental foundation trainees might be undertrained to make clinical decisions that are meaningful.

A minority of dentists indicated that their patients were of neutral opinion or dissatisfied with the decision making process. This could be due to a lack of confidence in complete denture techniques [32], or it could relate to the lack of routine NHS funding for implant retained prostheses in primary and secondary care [33]. For those unable to afford implants in the independent sector, some edentulous patients may, unfortunately, have no choice at all.

Comparing the responses to Question 8 in the current study to those of a group of North American prosthodontists [16], the results are fairly similar, although the percentage of clinicians advocating the consumerist model was nearly three times higher in the American study [16] than in the current study. This could be due to differences in the cultures of the UK and America with dental consumerism being slightly more developed in America [34,35].

The responses to Question 9 (Table 3) on being asked “What would you do if you were me?” produced a variety of responses. The majority of trainees would offer an answer, rather than expressing that their clinical concerns and preferences are likely to be different from the patient’s and declining to offer an answer. It could be viewed that declining to offer an answer is perhaps the most professional and ethical in that what the patient is really seeking by asking the question is the clinicians’ recommendation on the best plan. Perhaps this option was unpopular due to the pressure felt by clinicians to help patients. The majority (54.9%) of trainees indicated they would use their own values to answer the question, rather than their interpretation of the patient’s values (12.7%), or even using what they considered to be the average patient’s values (4.3%). It is acknowledged that 38% of the dentists would share their own clinical concerns and preferences to clarify differences with the patient’s circumstances, before offering an answer as if they were choosing for themselves. Few trainees indicated they would choose to answer the question as if they were the patient using their interpretation of the patient’s values. One must consider how accurately and comprehensively dentists can appraise patients’ values and preferences in a dental consultation appointment. Research from medicine has shown that surrogate decision makers, whether doctors, patient chosen relatives or next of kin show poor accuracy in predicting patient’s treatment preferences [36]. Question 9, which seeks a treatment recommendation, is subject to the self-other discrepancies seen in medical decision making. It has been shown [37] that doctors make more conservative treatment choices for their patients than for themselves, even if they accurately predicted that their patients would want a riskier treatment than the one they selected. Reasons behind this include the fear of legal consequences [38]. If these findings are applicable to dentistry, they would have relevance to the patients listening to recommendations from dentists, particularly since the patients were not aware of these discrepancies and thought that the decisions their doctors made for themselves would be similar to the decisions they made for their patients [37]. Question 9 was a realistic question that trainees most likely could have been asked in the past by patients, and so their response may well represent actual personal experience.

The dentists studied appeared to endorse the concept of shared decision making in the majority of Questions 3 to 8. It is of interest therefore, why in response to a more real-world scenario in Question 9, trainees were more likely to give a recommendation for treatment based on their own values than those of the patient. This same contradiction and discrepancy was seen in the American study [16]. A similar discrepancy has been noted between what factors dentists say are important in decision making in implant dentistry and those they actually use to make the decision to recommend implants to a patient [39]. The reasons behind the discrepancy found in this study are unclear. Further work, possibly of a qualitative or mixed methods nature in a real or simulated clinical environment would be required to obtain a more accurate, objective picture of trainees’ decision making styles.

The results showed that there was no strong evidence to suggest that there are gender differences in the decision making practices of the group of trainees studied. There was weak statistical evidence that males perceived edentulous patients’ values to be of less importance than females (*p* = 0.07). Males did indicate that the patient’s values were important, although perhaps not quite to the same extent as the females. This could be due to female dentists in general being more empathetic than their male counterparts. A critical review [8] of the physician–patient relationship found female physicians facilitate partnership and patient participation in the medical exchange more effectively than do male physicians. It is known that patients’ preferred decision making style or role is not static [26]. It varies within individuals and between individuals greatly, depending on factors such as the age and gender of the patient, gravity of the decision to be made, the clinical practice setting, the knowledge of the subject being discussed, trust in the dentist, time constraints, dissatisfaction with previous dental treatment, dental pain and the threat of wearing dentures [26]. Perhaps the ideal decision making style for dentists is an adaptive one, which varies according to the wishes of activity or passivity of the patient in decision making, whilst all the time respecting patient autonomy. The study did not aim to be representative of the entire UK Dental Foundation trainee population, and so the results of this study cannot be readily generalised to all UK trainees. There may be factors that affect trainees’ attitudes towards prosthodontic decision making which also affect their choice of region of the country and the associated Postgraduate Deanery of their Dental Foundation training programme. It is possible that trainees from the South West region sampled in this study are not representative of the whole of the UK trainee population in terms of their previous UK/overseas student status, university they qualified from, socio-economic status and ethnicity.

## 5. Conclusions

This study has provided some baseline findings in this little researched area of implant prosthodontics. The general consensus supporting shared decision making as an approach to decision making is encouraging, and is supportive of the UK dental schools’ ability to foster ethical and professional values among dentists. No gender differences being reported in the attitudes of dentists towards decision making is also encouraging, and can be used to inform undergraduate and dental foundation programme curriculum development in patient communication and the behavioural sciences.

## Figures and Tables

**Table 1:** Summary of participants’ responses to Questions 3 to 7.

Question Number	Median Response on Likert Scale (Interquartile Range)			*p*-Value (Mann-Whitney U Test of Association between Gender and Response)
	Overall	Male	Female	
**3**	3(2,4)	3(2,4)	3.5(2,4)	*p* = 0.8
**4**	1(1,2)	2(1,3)	1(1,2)	*p* = 0.07
**5**	3(3,4)	4(3,5)	3(2,4)	*p* = 0.3
**6**	3(2,4)	3(3,4)	3(2,4)	*p* = 0.3
**7**	2(2,3)	2(1,3)	3(2,3)	*p* = 0.6

**Table 2:** Participants’ responses to Question 8: “Ideally, how should clinicians and patients arrive at the optimal treatment option for the edentulous patient?”

Response Chosen by Participant	Percentage of Participants		
	Overall	Male	Female
No response given	4.3	3.7	4.6
(A) Choice of the best solution is fundamentally a technical decision; the clinician should make a strong recommendation to patients and seek their endorsement	0	0	0
(B) Choice of the best solution is partly a technical decision and partly based on the clinician’s preferences given what he/she knows about the patient	2.8	7.4	0
(C) Choice of the best solution results from negotiation between patients and clinicians after they have shared technical information as well as their values and preferences about the options	57.7	59.3	56.8
(D) Choice of the best solution is partly a technical decision and partly based on the patient’s informed preferences, regardless of the clinicians preferences	28.2	25.9	29.5
(E) Choice of the best solution is completely based on patient preferences; the clinician should only make sure the patient has adequate information about each option	7	3.7	9.1

**Table 3:** Participants’ responses to Question 9: “Which of the following would best describe your response to an edentulous patient who in response to your advice about treatment options asks, “What would you do if you were me?”

Response Chosen by Participant	Percentage of Participants		
	Overall	Male	Female
No response given	4.2	3.7	4.5
(A) Inform the patient that my clinical concerns and preferences are likely different from theirs and decline to offer an answer.	23.9	22.2	25.0
(B) Share my own clinical concerns and preferences to clarify differences with the patient’s circumstances, and offer an answer as if I was choosing for myself.	38.0	40.7	36.4
(C) Answer the question as if I was the patient and use my own values/ preferences to choose among the different treatment options.	16.9	11.1	20.5
(D) Answer the question as if I was the patient and use my interpretation of the patient’s values/preferences to choose among the different treatment options.	12.7	22.3	6.8
(E) Answer the question as if I was the patient and use my interpretation of the average patient’s values/preferences to choose among the different treatment options.	4.3	0	6.8
